# Should intravitreal dexamethasone implant in refractory diabetic macular edema be used as an adjuvant therapy or switch therapy?

**DOI:** 10.5935/0004-2749.2023-0229

**Published:** 2024-09-16

**Authors:** Semih Çakmak, Gokhan Demir, Uğur Tunç, Elmas Yuksel Sukun, Yusuf Berk Akbas, Abdullah Ozkaya, Ozgur Artunay, Gurkan Erdogan

**Affiliations:** 1 Department of Ophthalmology, Istanbul Faculty of Medicine, Istanbul University Istanbul, Turkey; 2 Department of Ophthalmology, Westeye Hospital, Erbil, Iraq; 3 Department of Ophthalmology, Eyup State Hospital, Istanbul, Turkey; 4 Alanya Training and Research Hospital, Antalya, Turkey; 5 Başakşehir Çam and Sakura Training and Research Hospital, Istanbul, Turkey; 6 Department of Ophthalmology, Memorial Sisli Hospital, Istanbul, Turkey; 7 Beyoglu Training and Research Hospital, Istanbul, Turkey

**Keywords:** Diabetic retinopathy, Macular edema/drug therapy, Dexamethasone/administration & dosage, Drug implants, Intravitreal injections, Ranibizumab/administration & dosage, Tomography, optical coherence, Endothelial growth factors

## Abstract

**Purpose:**

To compare the outcomes of intravitreal dexamethasone implant used as either an adjuvant or a switching therapy for diabetic macular edema in patients with poor anatomic response after three consecutive monthly injections of ranibizumab.

**Methods:**

This retrospective study included patients with diabetic macular edema who received three consecutive doses of ranibizumab as initial therapy and demonstrated poor response. A single dose of intravitreal de xamethasone implant was administered to these patients. The patients were divided into two groups according to the treatment modalities: the adjuvant therapy group, consisting of patients who continued treatment with ranibizumab injection after receiving intravitreal dexamethasone implant, and the switch therapy group, consisting of patients who were switched from ranibizumab treatment to intravitreal dexamethasone implant as needed. The main outcome measurements were best corrected visual acuity and central retinal thickness at baseline and at 3, 6, 9, and 12 months of follow-up.

**Results:**

In this study that included 64 eyes of 64 patients, the best corrected visual acuity and central retinal thickness values did not significantly differ between the groups at baseline and at 6 months of follow-up (p>0.05). However, at 12 months, the best corrected visual acuity values in the adjuvant and switch therapy groups were 0.46 and 0.35 LogMAR, respectively (p=0.012), and the central retinal thickness values were 344.8 and 270.9, respectively (p=0.007).

**Conclusions:**

In a real-world setting, it seems more reasonable to use intravitreal dexamethasone implant as a switch therapy rather than an adjuvant therapy for diabetic macula edema refractory to ranibizumab despite three consecutive monthly injections of ranibizumab. Patients switched to intravitreal dexamethasone implant were found to have better anatomic and visual outcomes at 12 months than those who continued ranibizumab therapy despite their less-than-optimal responses.

## INTRODUCTION

Diabetic macular edema (DME) is the leading cause of vision loss in patients with diabetes^(1^,^2)^. Its pathogenesis is multifactorial, arising from intricate mechanisms^(3)^. While factors such as retinal hypoxia, ischemia, and inflammation have been associated with DME, hyperglycemia remains the primary risk factor for diabetic retinopathy^(3)^.

Inflammation plays a pivotal role in the etiology of DME. The levels of inflammatory cytokines increase with increased inflammation in diabetic retinopathy and DME^(3^,^4)^. Consequently, it can be inferred that intravitreal antivascular endothelial growth factor (anti-VEGF) and intravitreal dexamethasone implants (IDI) play pivotal roles in the treatment of DME^(5^,^6^,^7)^.

While anti-VEGF treatments typically lead to anatomic and functional improvement, a notable proportion of eyes fail to exhibit improvement and may even experience increased vision loss^(8)^. Spectral-domain optical coherence tomography (OCT) is a frequently used imaging technique for diagnosing DME and monitoring treatment response^(9)^. The response in central retinal thickness (CRT) after anti-VEGF injection is also a predictor of long-term efficacy^(10)^. In DME treatment, intravitreal corticosteroid therapies are generally regarded as adjuncts or alternatives to anti-VEGF therapies rather than initial options owing to their potential ocular side effects such as elevated intraocular pressure and cataracts^(11)^. Thus, IDI may be considered for eyes with poor response to anti-VEGF therapy. According to the results from the Protocol I study of the Diabetic Retinopathy Clinical Research Network, no further improvement is observed when continuing ranibizumab treatment in patients with poor response to intravitreal ranibizumab treatment^(8)^. In such cases, a combination of treatments with steroids can be contemplated^(12)^.

The literature does not fully elucidate the timing of switching to another treatment and the subsequent treatment course in patients with sufficient response to ranibizumab. This study aimed to compare the outcomes of adjuvant therapy and switch therapy in patients initially treated with ranibizumab who exhibited poor anatomic response.

## METHODS

In this retrospective study conducted at the University of Health Sciences Turkiye, Beyoglu Eye Training and Research Hospital, the medical records of patients about intravitreal injections for DME administered between January and December 2019 were analyzed. Furthermore, the records of 107 previously untreated patients were scrutinized, and the study incorporated the data obtained from 64 eyes of 64 patients that met the inclusion criteria. The study was approved by the Beyoglu Training and Research Hospital and conducted in accordance with the principles of the Declaration of Helsinki. Before receiving intravitreal treatments, all patients provided both verbal and written informed consent after receiving the necessary explanations.

The study sample consisted of patients with DME who had not received any prior treatment. Cases Patients who received three consecutive doses of intravitreal ranibizumab as initial treatment were recorded. Those who had poor anatomic response to three doses of ranibizumab followed by IDI application and at least 12 months of follow-up were included in the final analysis. The included patients were divided into two groups: the adjuvant therapy group, consisting of patients who continued ranibizumab therapy after receiving intravitreal IDI, and the switch therapy group, consisting of patients who continued to receive IDI as needed.

The exclusion criteria were the detection of retinal ischemia in fundus fluorescein angiography, detection of vitreoretinal interface disease in OCT, history of previous intravitreal injection treatment, presence of proliferative diabetic retinopathy, follow-up period of <12 months, and macular edema resulting from any other condition. The demographic data of the patients were obtained by examining their records. In addition, the best corrected visual acuity (BCVA) and CRT values were recorded at baseline and at 3, 6, 9, and 12 months of follow-up. The number of injections administered to the patients during the follow-up period was also recorded. BCVA was tested at 20 feet using the Snellen chart. Detailed slit-lamp biomicroscopic and fundoscopic examinations were conducted in all patients. Intraocular pressure was measured using a Goldmann applanation tonometer.

The OCT device (Heidelberg Engineering, Heidelberg, Germany) was used for the CRT measurements. The mean thickness of the neurosensory retina in the central 1-mm diameter area, as calculated using the OCT mapping software, was defined as the CRT. Based on these measurements, patients with CRT of >300 µm were diagnosed with DME. In these patients, the poor anatomic response criteria were a decrease in CRT of less than 100 µm, an increase in CRT, or the absence of a foveal pit after three consecutive ranibizumab injections. Before treatment, fluorescein angiography (HRA-2; Heidelberg Engineering) assessments were conducted in all the patients. All examinations, except fluorescein angiography, were repeated during each follow-up visit.

Intravitreal injections were performed under topical anesthesia in a sterile room. The intravitreal injections consisted of ranibizumab (Lucentis® 0.5 mg/0.05 mL; Novartis, Basel, Switzerland) and the IDI (Ozurdex® 0.7 mg; Allergan Inc., Irvine, CA, USA). After cleaning the eye area with 10% povidone-iodine (Betadine®; Purdue Pharma, Stamford, CT, USA), 5% povidone-iodine was applied to the conjunctival sac. After the intravitreal injections, moxifloxacin drops (Vigamox®) were prescribed four times daily for 10 days. In all the patients, the treatment was started with a loading dose of three consecutive monthly injections of ranibizumab. After the initial loading treatment, the patients were reevaluated and IDI was applied to those with poor anatomic response. In the adjuvant therapy group, intravitreal ranibizumab injection was administered as needed following IDI application. In the switch therapy group, treatment continued with IDI as needed after the initial IDI. The primary outcome measure was the difference in the mean change in BCVA and CRT between the adjuvant and switch therapy groups.

Statistical analyses were conducted using the SPSS software for Windows (version 20.0, IBM Inc.). Before statistical analyses, the BCVA values were converted to the logarithm of the minimal angle of resolution (LogMAR) units. The normality of data distributions was evaluated using the Kolmogorov–Smirnov test. Continuous variables were expressed as mean and standard deviation, whereas categorical variables were expressed as absolute numbers and percentages. Repeated-measures analysis of variance with Bonferroni correction was employed to compare the BCVA and CRT values of the groups before and after the treatment. Independent samples *t*-test was used to compare the BCVA and CRT values between the groups. A p-value less than 0.05 was considered statistically significant.

## RESULTS

**Figure 1:**
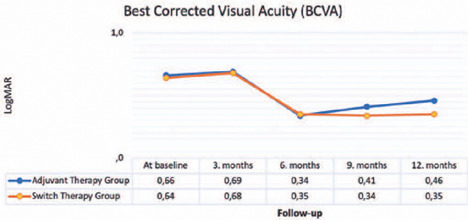
Mean BCVA values according to groups and follow-up examinations.

A total of 64 eyes of 64 patients were included in the study (30 eyes in the adjuvant therapy group and 34 eyes in the switch therapy group). In the adjuvant therapy group, 9 eyes were phakic and 21 eyes were pseudophakic. In the switch therapy group, 6 eyes were phakic and 28 eyes were pseudophakic. None of the phakic eyes in either group developed cataracts during the follow-up period. The groups were comparable in terms of age, sex, baseline BCVA, and baseline CRT (p>0.05 for all, Table 1). The mean BCVA values by group and follow-up visits are presented in figure 1. No statistically significant change was observed in BCVA from baseline to the third month in the adjuvant and switch therapy groups (p*=*0.181 and 0.118, respectively). However, a significant improvement in BCVA from baseline was found in both groups at the subsequent follow-up visits (p-values at 6, 9, and 12 months: 0.006, 0.011, and 0.013 for the adjuvant therapy group and 0.007, 0.014, and 0.015 for the switch therapy group). No statistically significant difference was observed in the comparison of the mean BCVA between the groups through 6^th^ month (p-values at baseline, 3 months, and 6 months: 0.412, 0.461, and 0.228, respectively). However, a significant improvement was observed in the mean BCVA in the switch therapy group at 9 and 12 months (p=0.021 and 0.012, respectively).

**Table 1:** Baseline parameters and demographic characteristics of the patients in both groups

Parameter (mean ± SD / n-%)	Adjuvant therapy group (n=30)	Switch therapy group (n=34)	p-value
Age (years)	58.51 ± 11.28	61.41 ± 9.19	0.821
Female/Male ratio	56.7%/43.3%	55.9%/44.1%	0.645
Baseline BCVA (LogMAR)	0.66 ± 0.12	0.64 ± 0.20	0.412
Baseline CRT (µm)	483.4 ± 11.1	488.2 ± 15.2	0.928

SD= standard deviation; BCVA= best corrected visual acuity; LogMAR= logarithm of the minimum angle of resolution; CRT= central retinal thickness.

**Figure 2:**
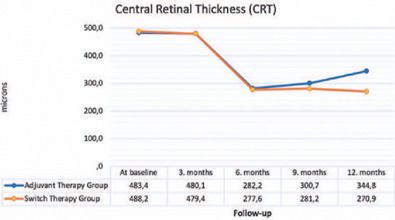
Mean CRT values according to groups and follow-up examinations.

The mean CRT values at follow-up according to the groups are shown in figure 2. At 3 months, no significant change was observed from baseline in the adjuvant and switch therapy groups (p=0.239 and 0.198, respectively). A significant decrease in the mean CRT from baseline was observed in the adjuvant therapy group (p-values at 6, 9, and 12 months: 0.001, 0.008, and 0.010, respectively) and the switch therapy group (p-values at 6, 9, and 12 months: 0.001, 0.001, 0.005, respectively). In the comparison of the mean CRT values between groups, no significant difference was observed in controls up to 6 months (*p*=0.928, 0.645, and 0.730, respectively). At 9 and 12 months of follow-up, the mean CRT values were found to be statistically significantly lower in the switch therapy group (p=0.012 and 0.007, respectively).

After the initial four intravitreal injections (three ranibizumab and one IDI) to all participants, an average of 5.65 ± 0.75 (range: 4–7) ranibizumab injections were administered in the adjuvant therapy group and 2.33 ± 0.50 (range: 2–3) IDI injections in the switch therapy group. After a total of 498 injections, no cases of injection-related endophthalmitis were reported.

## DISCUSSION

In our study, we evaluated the anatomic and visual outcomes of IDI application as a switch or adjuvant therapy in patients with DME who exhibited poor anatomic response to 3 months of continuous ranibizumab injections. To the best of our knowledge, our study is the first in the literature to compare the use of IDI as an adjuvant or switch therapy in patients with poor response to anti-VEGF in DME. In patients with DME exhibiting poor response or no response to ranibizumab, whether the next treatment is continued with either IDI or ranibizumab, IDI provides benefits in terms of visual and anatomic improvement. In our study, we found that continued IDI injections resulted in significantly improved anatomic and visual outcomes in these patients. In recent years, anti-VEGF drugs have been approved as first-line treatment for DME^(13)^. Due to the routine inclusion of these drugs as first-line treatment for DME, the number of anti-VEGF studies in the literature significant increased^(14^,^15)^. These studies reported that anti-VEGF agents such as ranibizumab, aflibercept, and bevacizumab are effective treatment options for DME^(16^,^17^,^18)^. However, these drugs do not exert the same effect in every patient^(19^,^20)^.

Although anti-VEGFs are the first-line treatment for DME, approximately 20%-25% of patients exhibit poor response to anti-VEGF therapy^(21^,^22)^. In their study, Usui-Ouchi et al.^(23)^ reported that baseline glycemic control and macular ischemia may be associated with response to intravitreal anti-VEGF injections. If the physician observes poor response to the anti-VEGF treatment being used, there are options to supplement the treatment with one of the other anti-VEGFs or with IDI. In addition to inhibiting VEGF pathways, IDI also reduces other inflammatory cytokines that play a pivotal role in the pathogenesis of DME. In this case, IDI is effective when there is no response to anti-VEGF therapy^(24^,^25)^. Hernandez Martínez et al.^(26)^ suggested that in eyes with poor response to anti-VEGF, continuing treatment for more than three doses adversely affects functional and anatomic outcomes. Touhami et al.^(27)^ reported that IDI increases the sensitivity of the retina to treatment in cases where the anti-VEGF response is poor.

In cases of poor response to anti-VEGF therapy, there are several options for anti-VEGF replacement or IDI supplementation. In a meta-analysis comparing the efficacy and safety of anti-VEGF and IDI as initial therapies for DME, He et al.^(28)^ found that these treatments were comparable in terms of visual improvement. However, they reported that IDI achieved better anatomic results with fewer injections at 6 months. Because of its ocular side effects, it can be assumed that IDI can be recommended as a first-line treatment in patients who do not respond to anti-VEGF agents and in selected patients who refuse frequent follow-up examinations^(29)^. In our clinical practice, we consider switching to IDI treatment in patients who do not respond to anti-VEGF injections. Demir et al.^(14)^ determined whether the use of IDI in cases of poor response to anti-VEGF treatment would lead to better results. The present study compared the results of switching to an IDI in patients with poor response to ranibizumab at 3 or 6 months. Early or late switch was found to be anatomically and functionally similar. However, early switching to IDI treatment may be more appropriate for patients’ comfort. It will also increase patient compliance. Thus, in our study, we included patients who exhibited poor response to ranibizumab, requiring a change in treatment after 3 months.

In cases of DME that is resistant to anti-VEGF therapy, a study reported that a single dose of IDI resulted in a statistically significant reduction in CRT at 6 months without any complications^(18)^. It also improved BCVA, although not statistically significant, and maintained good BCVA at 6 months. In our study group, there was no good visual and anatomic response to the initial three doses of ranibizumab in both groups. However, the BCVA and CRT values improved after IDI injection.

In our study, the average number of injections administered was lower in the switch therapy group than in the adjuvant therapy group. In addition to better anatomic and visual outcomes, fewer injections may provide better results in terms of economic burden and patient quality of life.

It has been reported in the literature that response to IDI may be better in the presence of biomarkers such as subfoveal neuroretinal detachment and hyperreflective retinal spots when macular examination is conducted with OCT^(9)^. The difference in the wavelengths of spectral domain and swept source (SS) OCT devices may affect the penetration of the rays. SS-OCT devices with longer wavelengths are superior to spectral domain OCT for revealing deeper lesions. Current research topics include the use of OCT angiography devices to detect microaneurysms and the use of images obtained from such devices in image processing programs(^30^).

This study has several limitations. First, we did not consider the OCT patterns. Second, the study has a retrospective design, short follow-up period, and relatively small sample size. Randomized controlled prospective studies are warranted to better elucidate the differences between treatments. Studies including aflibercept and faricimab will show the differences in the results of diffe rent anti-VEGF treatments. The strength of this study is that it is the first to compare adjuvant and switch treatments following IDI application in patients with poor response to consecutive ranibizumab injections.

In conclusion, visual and anatomic improvements occur when DME treatment is initiated with sequential ranibizumab injection as the initial approach. However, if there is no response, treatment is continued with adjuvant and switch treatment options. Our study demonstrated that better anatomic and functional outcomes were achieved when treatment was continued with IDI. Clinicians can consider this information when managing treatment, considering factors such as potential side effects and compliance.
